# Exploring the protective mechanism of Huangqishengmaiyin against anthracycline-induced cardiotoxicity in breast cancer with concomitant gut microbiota modulation

**DOI:** 10.3389/fcvm.2026.1818551

**Published:** 2026-10-12

**Authors:** Yixuan Wang, Yeying Jin, Jiali Han, Mingjia Ding, Haoqing Hou, Miaoyu Mei, Rui Yang

**Affiliations:** 1 Department of Breast Surgery, Shanxi Province Cancer Hospital, Shanxi Hospital Affiliated to Cancer Hospital, Chinese Academy of Medical Sciences, Cancer Hospital Affiliated to Shanxi Medical University, Taiyuan, China; 2 Fifth Clinical Medical College, Shanxi Medical University, Taiyuan, Shanxi, China; 3 First Clinical Medical College, Shanxi Medical University, Taiyuan, Shanxi, China; 4 College of Basic Medical Sciences, Shanxi Medical University, Taiyuan, Shanxi, China; 5 College of Public Health, Shanxi Medical University, Taiyuan, Shanxi, China

**Keywords:** anthracyclines, breast cancer, cardiac toxicity, gut microbiota modulation, Huangqishengmaiyin

## Abstract

**Objective:**

To elucidate the bioactive compounds and cardioprotective mechanism of Huangqishengmaiyin (HQSMY) against anthracycline-induced cardiotoxicity (AIC) in breast cancer with concomitant gut microbiota modulation.

**Methods:**

Myocardial fibrosis was quantified using Masson's trichrome staining. Hematoxylin and eosin staining was used for general histopathological evaluation. Ultra-high-performance liquid chromatography-tandem mass spectrometry (UHPLC-Q Exactive HFX) was employed to comprehensively identify blood components in HQSMY. The regulatory effect of HQSMY on the gut microbiota of AIC mice was assessed by 16S rRNA microbial sequencing. Network pharmacology and molecular docking mapped the compound-target-pathway network. Western blot was performed to detect key proteins in the predicted pathways, including JAK1, STAT3, and HIF-1*α*.

**Results:**

HQSMY reduced myocardial fibrosis rates, enhanced gut microbiota diversity, abundance and health indices. Meanwhile, HQSMY increased the abundance of anti-inflammatory bacteria like Bacteroidetes and restored normal metabolic pathways in multiple gut microbes, including glycolysis. 42 blood components were identified, exhibiting antioxidant, anti-inflammatory, and gut microbiota-regulating activities. Network pharmacology analysis revealed that the JAK-STAT and HIF-1 signaling pathways are key mechanisms. Enrichment analysis focused on energy metabolism and inflammatory response. Western blot results confirmed that HQSMY significantly downregulated JAK1 and HIF-1*α* protein expression; STAT3 was decreased without reaching statistical significance.

**Conclusion:**

The components of HQSMY exhibit anti-inflammatory effects in myocardial tissue, meanwhile enhancing beneficial gut bacteria in intestine. The regulatory effect of HQSMY may be exerted through JAK-STAT and HIF-1 pathways, as confirmed by altered expression of key proteins in these pathways.

## 1 Introduction

Breast cancer is the most commonly diagnosed cancer and the leading cause of cancer-related death among women worldwide (1). Currently, anthracycline drugs serve as the cornerstone chemotherapy for malignant breast tumors. However, their use in breast cancer treatment can lead to irreversible anthracycline-induced cardiotoxicity (AIC). The 2022 international guidelines on cardiac oncology define AIC based on the following criteria: a relative decrease in left ventricular ejection fraction (LVEF) > 15% or cTnI/cTnT > 99%, BNP ≥ 35 pg/mL, or NT-proBNP ≥ 125 pg/mL (2). Manifestations include heart failure, myocarditis, vascular toxicity, arrhythmias, and left ventricular dysfunction, posing a significant threat to patient survival (3). Notably, this drug-induced cardiomyopathy often prevents patients from completing full chemotherapy, ultimately leading to tumor progression. Clinically, anthracyclines are often combined with dexrazoxane (Dex) to prevent cardiotoxicity. However, Dex is associated with severe hematological adverse effects, including leukopenia, neutropenia, and thrombocytopenia, and requires bone marrow transplantation in some cases (4). Therefore, identifying effective strategies to treat AIC has become an urgent priority in breast cancer therapy.

AIC falls under Traditional Chinese Medicine (TCM) syndromes such as palpitations and chest-fluid obstruction (5). Sheng-Mai Yin (SMY), a classic TCM formulation for tonifying qi and nourishing yin that originated from YiXueQiYuan during the Yuan Dynasty, consists of three medicinal herbs: *Radix* Ginseng Rubra (Panax ginseng C. A. Mey.), *Ophiopogon* japonicus (Thunb.) Ker Gawl., and *Schisandra* chinensis (Turcz.) Baill. It is widely used in cardiovascular diseases with qi-yin deficiency syndrome (6). The efficacy of SMY on AIC has been evaluated by meta-analysis, showing that it can improve ST-T segment changes and creatine kinase isoenzyme, relieve prolonged QT interval, sinus tachycardia, and premature ventricular contractions in patients with AIC (7). With the evolution of science, it has progressively diversified into various dosage forms, while its prescription formulation has also been refined and modified. Huangqishengmaiyin (HQSMY) is an enhanced version of SMY, replacing *Radix* Ginseng Rubra with *Pseudostellaria* heterophylla (Miq.) Pax and incorporating *Astragalus* mongholicus Bunge as the sovereign drug. Given that *Astragalus* mongholicus has been demonstrated to alleviate myocardial fibrosis, pathological myocardial injury, and to inhibit oxidative stress and inflammatory responses, its incorporation into SMY can enhance the cardioprotective effects (8). Astragaloside IV reduces inflammatory cell infiltration in the myocardium while decreasing NLRP3 inflammasome expression and its downstream caspase-1, IL-18, and IL-1β protein levels (9). *Pseudostellaria* heterophylla, milder in nature than *Radix* Ginseng Rubra, is particularly suitable for patients with post-chemotherapy debility. It exhibits immunomodulatory, hypoglycemic, anti-inflammatory, cytoprotective, and tyrosinase-inhibiting effects (10). *Ophiopogon* japonicus is commonly used to treat palpitations and startle responses due to heart yin deficiency. Polysaccharides from Ophiopogon japonicus reduce doxorubicin (Dox)-induced myocardial ferroptosis by activating the Nrf2/GPX4 signaling pathway and mitigating iron accumulation (11). Schisandra regulates cardiovascular circulation, exhibiting significant blood pressure-elevating effects in circulatory failure patients. Schisandrin B prevents AIC by enhancing the glutathione redox cycle (12). Therefore, we speculate that HQSMY may exert superior clinical efficacy compared to SMY in the treatment of AIC.

Notably, numerous components in HQSMY have been demonstrated to exert cardioprotective effects through the gut microbiota and its metabolites. For example, Astragalus polysaccharides stimulate the proliferation of beneficial bacterial genera, such as Allobaculum and Lactobacillus, thereby strengthening the integrity of the intestinal barrier (13). TCM theory holds that the heart and small intestine are paired organs, closely linked in their meridian pathways and physiological functions. Modern gut microbiota research has revealed a close relationship between the cardiovascular system and the intestinal microecology. A balanced intestinal microecology promotes cardiovascular homeostasis. Conversely, an imbalanced gut microbiota can exert pathological effects on the cardiovascular system. This bidirectional regulation suggests a potential association between gut microbiota and cardiac function (14).

Given the intricate composition and multi-target nature of TCM, the bioactive components of HQSMY, which are absorbed into the bloodstream via the intestinal barrier following oral administration, were identified using UHPLC-Q Exactive HF-X. Meanwhile, this study evaluated the regulatory effect of HQSMY on the gut microbiota of AIC model mice through 16S rRNA microbial sequencing. Combined with network pharmacology methods, key signaling pathways and potential target molecules were identified. This study aims to elucidate the active components and mechanisms of HQSMY in treating AIC with concurrent assessment of gut microbiota changes.

## 2 Materials

### 2.1 Samples and instruments

Methanol, acetonitrile, formic acid, and isopropyl alcohol were purchased from ANPEL. All solvents were LC-MS grade. Ultra-pure water was prepared in-house using a Milli-Q water purification system (Millipore, Bedford, MA, USA). Masson's trichrome staining kit was purchased from Solarbio (Beijing, China). Primary antibodies against JAK1 (Proteintech, Cat No. 27284-1-AP, rabbit polyclonal, 1:1000), STAT3 (Proteintech, Cat No. 10253-2-AP, rabbit polyclonal, 1:1000), HIF-1*α* (Active Motif, Clone ESEE122, mouse monoclonal, 1:1000), and *β*-actin (Proteintech, Cat No. 81115-1-RR, rabbit recombinant, 1:2000) were used. Horseradish peroxidase-conjugated goat anti-rabbit IgG (H + L) secondary antibody (1:5000, #7074) and horseradish peroxidase-conjugated goat anti-mouse IgG (H + L) secondary antibody (1:5000, #7076) were purchased from Cell Signaling Technology (Danvers, MA, USA).

### 2.2 Drug preparation

HQSMY is composed of 12 g Ophiopogon japonicus (Thunb.) Ker Gawl. (Mai Dong), 6 g Schisandra chinensis (Turcz.) Baill. (Wu Wei Zi), 15 g Pseudostellaria heterophylla (Miq.) Pax (Tai Zi Shen), and 30 g Astragalus mongholicus Bunge (Huang Qi), purchased from the Traditional Chinese Medicine Pharmacy of Shanxi Cancer Hospital. HQSMY crude drugs were soaked in water for 30 min and decocted twice: the first decoction used 10 times the weight of water and simmered for 1 h; the liquid was filtered. The second decoction used 8 times the weight of water and simmered for 1 h; the filtered liquids were combined. The mixture was concentrated using a rotary evaporator, then placed in a freeze-dryer to produce lyophilized powder. The clinical human dose (63 g crude drugs/day for a 60-kg adult) was converted to the mouse equivalent dose using body surface area normalization (approximately 10-fold) (15). Administered doses were calculated as lyophilized extract (extraction yield: 16.2%, w/w). The medium dose (1.7 g/kg/day) corresponded to the clinical equivalent dose, and the high dose (3.4 g/kg/day) was 2-fold higher. For use, the lyophilized powder was dissolved in distilled water and administered by oral gavage once daily at the indicated doses.

### 2.3 Animals

Twenty-five SPF female BALB/c mice, 6 weeks old and weighing 18–20 g, were purchased from Shanghai Sileke Experimental Animal Co., Ltd. The living conditions were maintained in accordance with SPF standards, featuring a 12-hour light/12-hour dark cycle, an ambient temperature of 22 °C ± 2 °C, humidity levels of 50% ± 10%, and free access to water. Doxorubicin (Dox): GC16994; Dexrazoxane (Dex): HY-B0581. The 4T1 cells were obtained from the Center for Fine Resources at the Shanghai Institutes for Biological Sciences, which is affiliated with the Chinese Academy of Sciences.

### 2.4 Databases and software

GraphPad Prism 11.0; GeneCards (https://www.genecards.org); HERB (http://herb.ac.cn); SwissADME (http://www.swissadme.ch); SwissTargetPrediction (http://swisstargetprediction.ch); UniProt (https://www.uniprot.org); STRING (https://string-db.org); KEGG (https://www.genome.jp/kegg); TTD (https://db.idrblab.net/ttd); DrugBank (https://go.drugbank.com); PubChem (https://www.ncbi.nlm.nih.gov/pccompound); Omicshare (https://www.omicshare.com); PDB (https://www.rcsb.org); Cytoscape 3.7.2; CytoNCA software; MCODE application; PyMOL-2.5.2 software; AutoDockTools-1.5.7 software; Majorbio Bio-Pharm Technology cloud platform.

## 3 Methods

### 3.1 Animal model grouping and administration

Twenty-five BALB/c mice were acclimatized for one week and then randomly divided into five groups: Control, Model, HQSMY-M (1.7 g/kg/day), HQSMY-H (3.4 g/kg/day), and Positive Control-Dex (*n* = 5). Under aseptic conditions, 2 × 10⁵ 4T1 cells were injected into the second pair of right mammary fat pads of mice in the four groups (excluding the Control group) to establish a triple-negative breast cancer mouse model. Two days after tumor cell injection, mice in the four groups received intraperitoneal injections of Dox at 10 mg/kg/week to induce cardiotoxicity. The HQSMY groups were gavage-administered with the drug solution at 1.7 or 3.4 g/kg/day, while the Dex group received 100 mg/kg/week intraperitoneally. After 28 days, mice were euthanized, and cardiac tissue was harvested, fixed in 4% paraformaldehyde, embedded, and stained with H&E for general histopathological evaluation and Masson's trichrome for collagen deposition and fibrosis quantification. Masson's trichrome staining was performed on paraffin-embedded cardiac tissue sections (5 μm) according to the manufacturer's protocol. Collagen fibers were stained blue, while muscle fibers and cytoplasm were stained red. Images were captured under a light microscope (Olympus BX53, 200×). The percentage of fibrotic area was quantified using ImageJ software.

Mice were monitored twice daily for signs of distress throughout the study. Predefined humane endpoints included: (1) > 15% body weight loss sustained for 72 h, (2) severe lethargy or immobility, (3) dyspnea or cyanosis, (4) self-mutilation or prolonged convulsions. Animals reaching any of these endpoints were humanely euthanized by exposure to 1%–3% isoflurane until loss of the righting reflex (typically within 2 min), followed by cervical dislocation or bilateral pneumothorax to ensure death.

Subsequently, fecal microbiota 16S rRNA sequencing analysis was performed on 20 mice (*n* = 5 per group) from the Control, Model, HQSMY (1.7 g/kg/day), and Dex groups to examine gut microbiota changes under HQSMY intervention. Simultaneously, plasma samples were collected from 10 mice (*n* = 5 per group) in the Control and HQSMY (1.7 g/kg/day) groups. These samples were compared with herbal medicine samples (TCM-HQSMY) to identify HQSMY components entering the bloodstream. The medium dose (1.7 g/kg/day) was selected for 16S rRNA sequencing and plasma metabolomics based on comparable efficacy observed in the fibrosis assay and in accordance with the 3R principles.

### 3.2 Identification of HQSMY blood components

#### 3.2.1 Sample preparation and extraction

Plasma samples were thawed slowly at 4 °C. An aliquot (1.0 mL) was transferred to a freeze-drying centrifuge tube, and an equal volume of extraction solution (methanol-acetonitrile, 1:1, v/v) containing 4 μg/mL L-2-chlorophenylalanine internal standard was added. The mixture was homogenized for 60 s and extracted by low-temperature ultrasonication for 30 min (twice), then incubated for 1 h to precipitate protein at −20 °C, followed by centrifugation at 12,000 rpm for 20 min at 4 °C. The supernatant was dried under vacuum, and 200 μL of 30% acetonitrile solution was added to re-dissolve. After homogenization and centrifugation at 14,000 rpm for 15 min at 4 °C, the supernatant was collected for detection. Quality control samples were prepared by mixing equal volumes of the supernatants from the HQSMY and Control groups, followed by centrifugation to obtain pooled samples.

### 3.3 Mass spectrometry detection method

The liquid chromatography parameters are as follows. Column: Waters HSS T3 (100 × 2.1 mm, 1.8 μm); Mobile phase: Phase A is ultrapure water solution (containing 0.1% formic acid), Phase B is acetonitrile solution (containing 0.1% formic acid); Flow rate: 0.3 mL/min; Column temperature: 40 °C; Injection volume: 2 μL; Elution gradient: 0 min A/B (100:0, v/v), 1 min A/B (100:0, v/v), 12 min A/B (5:95, v/v), 13 min A/B (5:95, v/v), 13.1 min A/B (100:0, v/v), 17 min A/B (100:0, v/v). Throughout the analysis, samples were maintained at 4 °C in the autosampler. Mass spectrometry conditions: Electrospray ionization source, sheath gas 40 arb, auxiliary gas 10 arb, ion spray voltage +3000 V/−2,800 V, temperature 350 °C, ion transfer tube temperature 320 °C. Scan mode: Full-ms-ddMS2, scan type: positive/negative ions. The primary mass spectrum scan range was 70–1,050 Da, with primary resolution of 70,000 and secondary resolution of 17,500.

#### 3.3.1 Data preprocessing

Raw data underwent preliminary processing using Progenesis QI for baseline filtering, peak identification, retention time correction, peak matching, and peak alignment. This yielded a data matrix containing mass-to-charge ratios, retention times, and peak intensities. Subsequently, qualitative analysis of metabolites in the samples was completed by matching against Huijun Bio's proprietary secondary mass spectrometry database of Chinese herbal medicines. Among the compounds identified through QI database searches, only those with a total score ≥50 were retained as identified components of HQSMY.

### 3.4 Fecal microbiota 16S rRNA sequencing

Bacterial DNA was extracted from fecal samples using the PowerSoil DNA Isolation Kit, and the V3-V4 region of the bacterial 16S rRNA gene was amplified using primers 338F and 806R. Pair-end sequencing of purified amplicons was performed by Majorbio Bio-Pharm Technology (Shanghai, China) on an Illumina MiSeq PE300 platform (Illumina, San Diego, USA). Data analysis and visualization were conducted on the Majorbio BioCloud platform. All obtained raw sequence datasets have been uploaded to the NCBI Sequence Read Archive (SRA) with the accession number PRJNA1372457.

### 3.5 Network pharmacology-based prediction of HQSMY's AIC inhibition mechanism

Blood-penetrating component targets were collected via PubChem and SwissTargetPrediction. Genes associated with breast cancer and AIC were obtained from the GeneCards database. The STRING database was used to construct a protein-protein interaction network. Cytoscape 3.7.2 performed network topology analysis. The MCODE program calculated pivotal targets. Target IDs were converted to gene IDs via the Omicshare website. Gene Ontology (GO) and Kyoto Encyclopedia of Genes and Genomes (KEGG) enrichment analyses were subsequently performed. Cytoscape 3.7.2 was used to construct a “Chinese medicine-compound-target-pathway-disease” interaction network and generate a Sankey diagram. Molecular docking validation followed: structural formulas were downloaded from PubChem, while protein structures were obtained from the PDB database. Water molecules were removed using PyMOL-2.5.2 software. Binding energy values were obtained using AutoDockTools-1.5.7. Visualization was performed with PyMOL-2.5.2.

### 3.6 Western blot analysis

Cardiac tissue samples were lysed in RIPA buffer containing protease and phosphatase inhibitors. Protein concentrations were determined using the bicinchoninic acid assay kit. Equal amounts of protein (30 μg) were separated by 10% SDS-PAGE and transferred to PVDF membranes. Membranes were blocked with 5% bovine serum albumin for 1 h at room temperature, then incubated overnight at 4 °C with primary antibodies against JAK1, STAT3, HIF-1*α*, and *β*-actin. After washing with TBST, membranes were incubated with the corresponding horseradish peroxidase-conjugated secondary antibodies [goat anti-rabbit or goat anti-mouse IgG (H + L), 1:5,000] for 1 h at room temperature. Protein bands were visualized using an enhanced chemiluminescence reagent and captured with a chemiluminescence imaging system. Band intensities were quantified using ImageJ software and normalized to *β*-actin. Antibody dilutions are detailed in Section 2.1.

### 3.7 Statistical methods

Data processing and analysis were conducted using GraphPad Prism 11.0. All data are expressed as mean ± SD. Independent samples t-tests or one-way ANOVA were applied. A *P* < 0.05 was considered statistically significant.

## 4 Results

### 4.1 HQSMY reduced myocardial fibrosis rate of the AIC mice

To investigate the therapeutic potential of HQSMY against AIC, we established a Dox-induced cardiotoxicity model in female BALB/c mice bearing 4T1 breast tumors. The experimental workflow is depicted in Figure 1A. Histopathological analysis of H&E-stained myocardial sections revealed marked structural damage in the Model group compared to the Control group (Figure 1B). Masson's trichrome staining further demonstrated extensive collagen deposition (blue staining) in the Model group, reflecting severe Dox-induced myocardial fibrosis (Figure 1C). Remarkably, treatment with HQSMY (both medium and high doses) or Dex substantially reduced collagen accumulation. Quantitative analysis of the fibrotic area confirmed that the myocardial fibrosis rate was significantly decreased in the HQSMY-M, HQSMY-H, and Dex groups relative to the Model group (Figure 1D).

**Figure 1:**
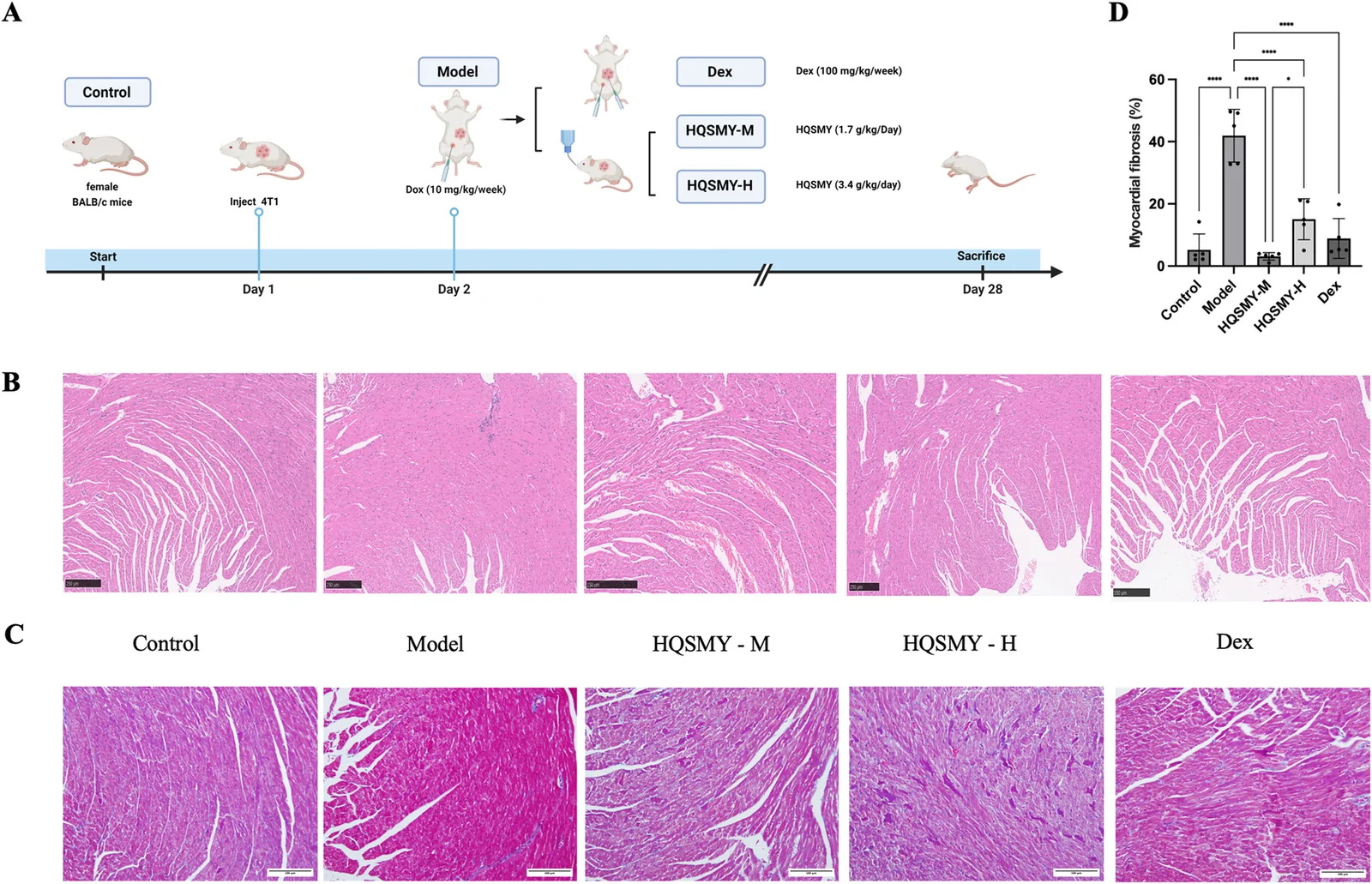
HQSMY reduced the incidence of doxorubicin-induced myocardial fibrosis. **(A)** Schematic illustration of the experimental design. Female BALB/c mice were inoculated with 4T1 cells on Day 1 and treated with doxorubicin (Dox, 10 mg/kg/week) from Day 2 to establish the cardiotoxicity model. Mice in the HQSMY-M and HQSMY-H groups were orally administered HQSMY at 1.7 g/kg/day and 3.4 g/kg/day, respectively, from Day 2. The Dex group received dexrazoxane (100 mg/kg/week). All mice were sacrificed on Day 28. **(B)** Representative images of hematoxylin and eosin (H&E) staining of myocardial tissue from each group. Scale ba*r* = 250 μm. **(C)** Representative images of Masson's trichrome staining of myocardial tissue from each group. Collagen fibers are stained blue. Scale ba*r* = 100 μm. **(D)** Quantitative analysis of myocardial fibrosis area based on Masson's trichrome staining. *P < 0.05, **P < 0.01, ***P < 0.001.

### 4.2 Analysis of HQSMY components and plasma components following administration

To determine the composition of HQSMY, we employed UHPLC-Q Exactive HFX for its component analysis. The base peak chromatogram of TCM-HQSMY is shown in Figure 2A. With a fragmentation score greater than or equal to 50 points, a total of 546 compounds in TCM-HQSMY were annotated. These included 145 lipids and lipid-like molecules, 99 phenylpropanoids and polyketides, 54 organic oxygen compounds, 49 organoheterocyclic compounds. 33 organic acids and derivatives, 18 benzenoids, 10 nucleosides, nucleotides, and analogues, 9 lignans, neolignans and related compounds, 4 alkaloids and derivatives, 2 organic nitrogen compounds, and 123 other compounds (Supplementary Table S1). Figure 2B shows the percentage of compound quantities by class in TCM-HQSMY.

**Figure 2:**
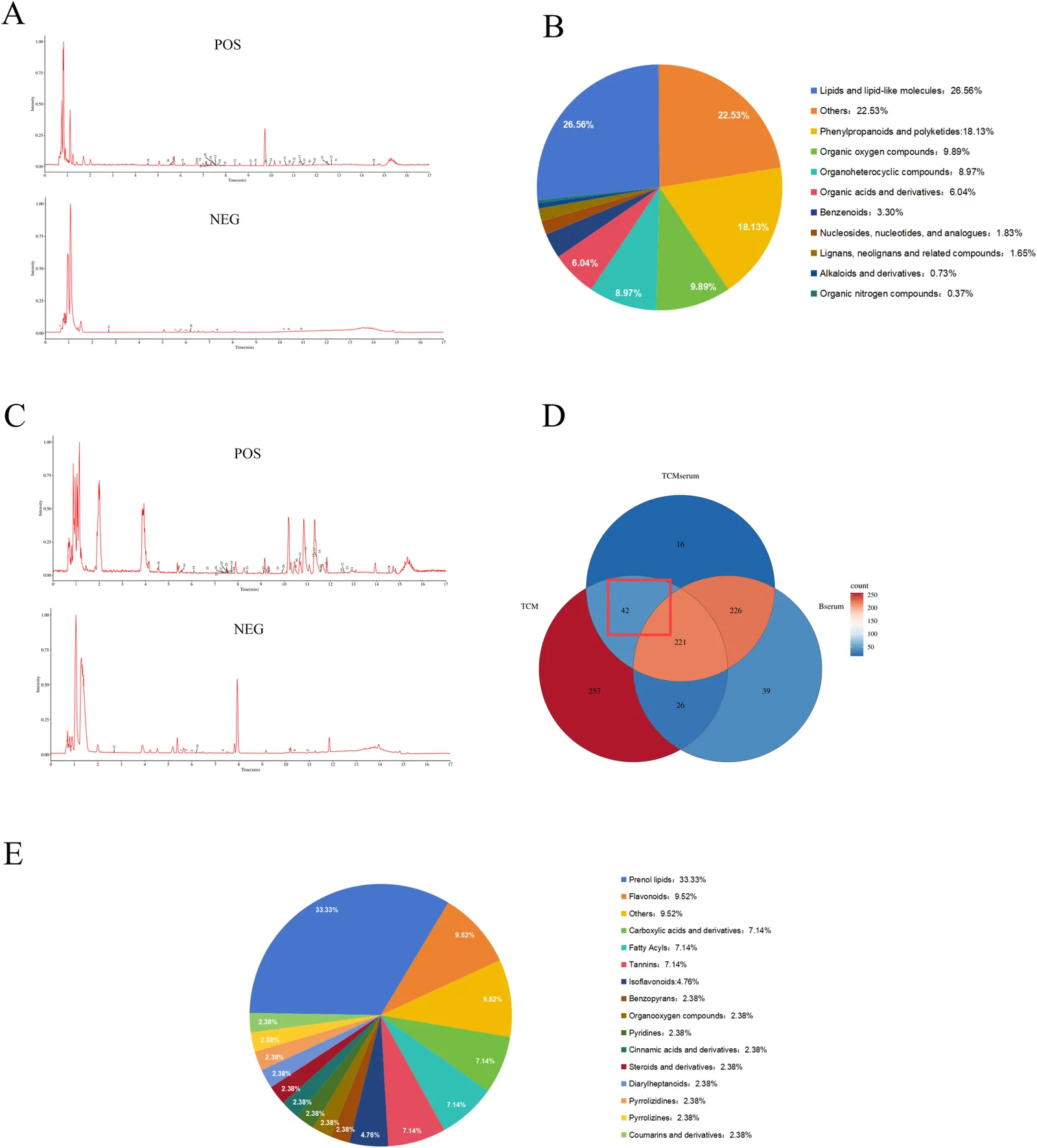
Analysis of HQSMY components. **(A)** BPC of TCM-HQSMY. **(B)** Percentage distribution of compound quantities by class in TCM-HQSMY. **(C)** BPC of HQSMY plasma samples. **(D)** Venn diagram. **(E)** Blood component classification.

The chromatograms of HQSMY plasma samples are shown in Figure 2C. Using Venn diagram analysis of plasma and herbal medicine components, 42 absorbed components were identified (Figure 2D). The pie chart classifying absorbed components according to Chinese subcategories is shown in Figure 2E. Detailed information on the 42 absorbed components is provided in Table 1. Among these, flavonoids (16), tannins (17), isoflavonoids (18), coumarins and derivatives (19) have been verified to have anti-inflammatory activity. This may be the key reason why HQSMY reduced myocardial inflammation and alleviated AIC by improving both intestinal and myocardial conditions.

**Table 1:** HQSMY components in the plasma samples.

Class A	Class B	Number	Components	Adducts	m/z	Formula	Class C
Lipids and lipid-like molecules	Prenol lipids	1	Eucommiol	2[M+K]^+^	415.174	C_9_H_16_O_4_	Monoterpenoids
		2	Calealactone C	[M+H-2H2O]^+^	371.148	C_21_H_26_O_8_	Terpene lactones
		3	Teucroxide	[M+Na]^+^	401.159	C_20_H_26_O_7_	Terpene lactones
		4	Wedeliatrilolactone A	[M+H-2H2O]^+^	417.190	C_23_H_32_O_9_	Terpene lactones
		5	8alpha-Methacryloyloxybalchanin	[M-H2O-H]^-^	313.145	C_19_H_24_O_5_	Terpene lactones
		6	Caesalmin E	[M+Na]^+^	515.227	C_26_H_36_O_9_	Diterpenoids
		7	Phorbol 13-acetate	[M-H2O-H]^-^	387.182	C_22_H_30_O_7_	Diterpenoids
		8	Chaetopenoid A	[M+Na]^+^	437.195	C_24_H_30_O_6_	Sesquiterpenoids
		9	Notopterol	[M+CH3OH+H]^+^	387.179	C_21_H_22_O_5_	Terpene lactones
		10	Tanshinone IIA	[M+H]^+^, [M+Na]^+^	295.132	C_19_H_18_O_3_	Diterpenoids
		11	Caleurticolide 2-methylacrylate	[M+H-2H2O]^+^	397.164	C_23_H_28_O_8_	Terpene lactones
		12	Dihydrotanshinone I	[M+H]^+^	279.101	C_18_H_14_O_3_	Diterpenoids
		13	Methyl 6-hydroxyangolensate	[M+FA-H]^-^	531.223	C_27_H_34_O_8_	Triterpenoids
		14	Yomogiartemin	[M+H-2H2O]^+^	301.107	C_17_H_20_O_7_	Terpene lactones
	Fatty Acyls	15	Myriocin	[M+2Na-H]^+^	446.252892825416	C_21_H_39_NO_6_	Fatty acids and conjugates
		16	Virginiaebutanolide C	[M+FA-H]^-^	261.134	C_11_H_20_O_4_	Fatty alcohols
		17	Methyl hydrogen azelate	[M+FA-H]^-^	247.119	C_10_H_18_O_4_	Fatty acids and conjugates
	Steroids and derivatives	18	Juncuenin D	[M+CH3OH+H]^+^	315.158431165091	C_18_H_18_O_3_	Oxosteroids

Class A	Class B	Number	Metabolite	Adducts	m/z	Formula	Class C
Phenylpropanoids and polyketides	Flavonoids	19	Wogonoside	[M-H]^-^	459.094	C_22_H_20_O_11_	Flavonoid glycosides
		20	Tectochrysin	[M+H]^+^	269.080	C_16_H_12_O_4_	O-methylated flavonoids
		21	Homoplantaginin	[M+H]^+^	463.123	C_22_H_22_O_11_	Flavonoid glycosides
		22	Herbacetin	[M+ACN+H]^+^	344.075	C_15_H_10_O_7_	Flavones
	Tannins	23	Heteroclitin G	[M+H-H2O]^+^, [M+H]^+^	401.159	C_22_H_24_O_7_	Hydrolyzable tannins
		24	Schisandrin B	[M+H]^+^, [M+Na]^+^, [M+NH4]^+^	401.195	C_23_H_28_O_6_	Hydrolyzable tannins
		25	Angeloylgomisin H	[M+H-H2O]^+^, [M+NH4]^+^, [M+K]^+^, [M+Na]^+^, [M+H]^+^	483.237	C_28_H_36_O_8_	Hydrolyzable tannins
	Isoflavonoids	26	Dehydroglyasperin C	[M+CH3OH+H]^+^	387.179	C_21_H_22_O_5_	O-methylated isoflavonoids
		27	calycosin	[M+H]^+^	285.075	C_16_H_12_O_5_	O-methylated isoflavonoids
	Cinnamic acids and derivatives	28	Xanthohumol L	[M+CH3OH+H]^+^	403.174	C_21_H_22_O_6_	Hydroxycinnamic acids and derivatives
	Diarylheptanoids	29	Isotriuvaretin	[M+H-2H2O]^+^	555.220	C_37_H_34_O_7_	Linear diarylheptanoids
	Coumarins and derivatives	30	7-Amino-4-methylcoumarin	[M+Cl]^-^	268.038	C_12_H_11_NO_4_	-
Organoheterocyclic compounds	Pyridines	31	Nicotinic acid	[M+FA-H]^-^	168.030	C_6_H_5_NO_2_	Pyridinecarboxylic acids
	Pyrrolizidines	32	Procerine	[M+NH4]^+^	289.175	C_13_H_21_NO_5_	-
	Pyrrolizines	33	Crosemperine	[M+Cl]^-^	403.177	C_19_H_30_NO_6_+	-
	Benzopyrans	34	Eupatoriochromene	[M+H]^+^	219.101	C_13_H_14_O_3_	1-benzopyrans
	Carboxylic acids and derivatives	35	8alpha-Tigloyloxyhirsutinolide 13-O-acetate	[M+H-2H2O]^+^	385.164	C_22_H_28_O_8_	Tricarboxylic acids and derivatives
		36	2,6-Diaminoheptanedioic acid	[M-H]^-^	189.088	C_7_H_14_N_2_O_4_	Amino acids, peptides, and analogues
		37	L-Homocystine	[M+H]^+^	269.065	C_8_H_16_N_2_O_4_S_2_	Amino acids, peptides, and analogues
Organic oxygen compounds	Organooxygen compounds	38	Simmondsin	[M+ACN+H]^+^	417.190	C_16_H_25_NO_9_	Carbohydrates and carbohydrate conjugates
Others	Others	39	Schisantherin A	[M+NH4]^+^	554.238	C_30_H_32_O_9_	-
		40	Homoharringtonine	[M+CH3OH+H]^+^	578.295	C_29_H_39_NO_9_	-
		41	Lentinan	[M-2H]^-^	575.185	C_42_H_72_O_36_	-
		42	Licoagrochalcone B	[M+CH3OH+H]^+^	369.169	C_21_H_20_O_4_	-

### 4.3 HQSMY improved gut microbiota in AIC mice

#### 4.3.1 HQSMY altered intestinal microbe diversity and microbial health index in AIC mice

We performed diversity testing from different dimensions, including *α*-diversity and *β*-diversity analysis. *α*-Diversity showed that compared to the Control group, the Model group exhibited reduced Shannon and Chao indices. Following intervention, the Shannon and Chao indices increased in both the HQSMY and Dex groups (Figures 3A,B). Figure 3C illustrates the reasonableness of sequencing data volume via a dilution curve; Figure 3D displays species richness at the operational taxonomic unit level across the four groups, with the HQSMY group's richness closest to the Control group. *β*-Diversity analysis revealed that the Control group and both intervention groups are distant from the Model group, while the HQSMY and Dex groups are closer (Figure 3E). Compared to the Model group, the HQSMY group exhibited a higher microbial health index (Figure 3F) and a lower dysbiosis index (Figure 3G).

**Figure 3:**
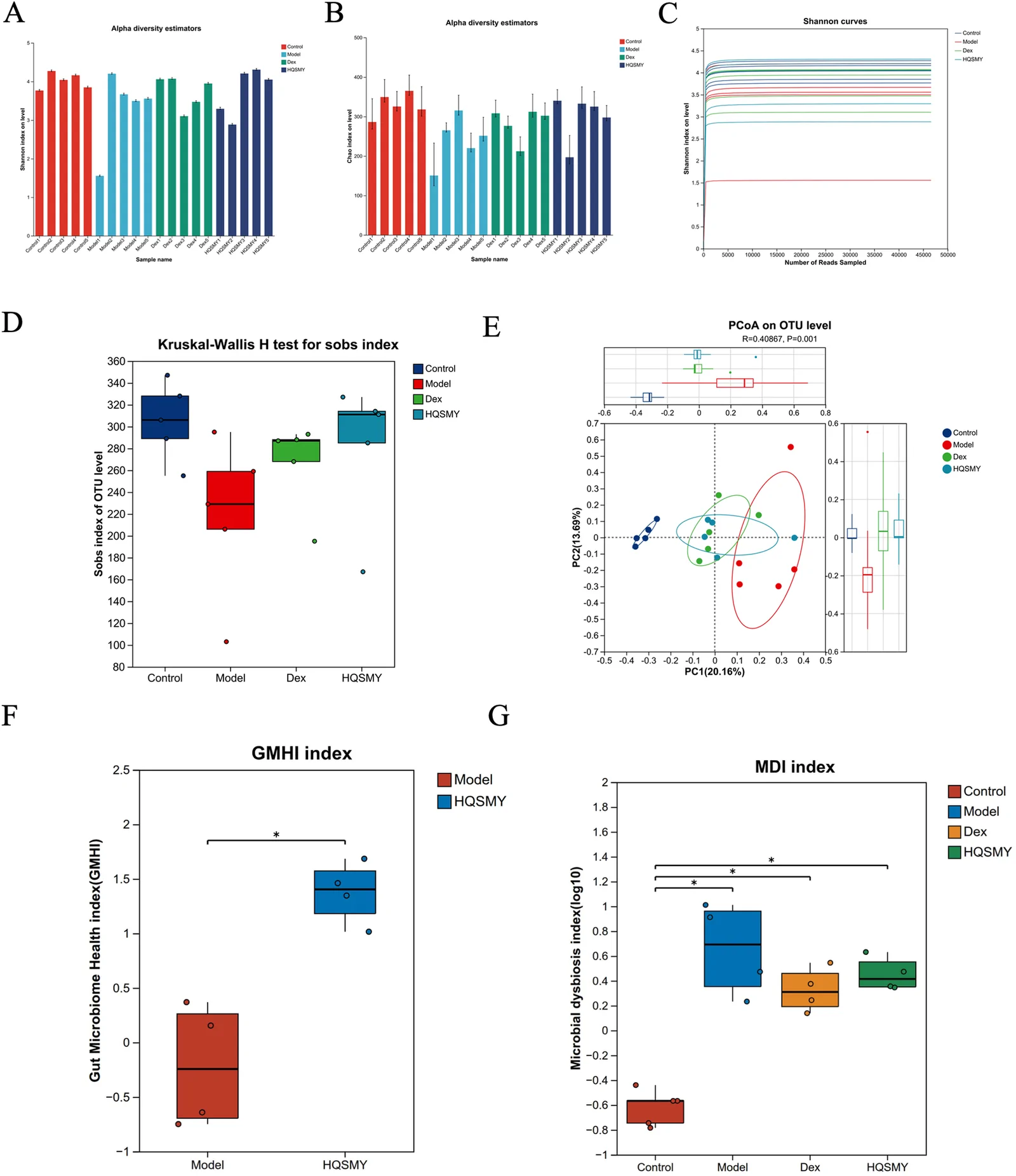
Diversity analysis and microbial community characterization indices. **(A)** α-Diversity analysis: Shannon index reflecting both species richness and evenness **(B)** α-Diversity analysis: Chao index, reflecting species richness (estimated total number of species) **(C)** Dilution curve analysis **(D)** Inter-group difference test (Kruskal–Wallis H-rank test) **(E)** PcoA analysis **(F)** Microbial health index **(G)** Microbial dysbiosis index.

#### 4.3.2 HQSMY adjusted the structure of gut microbes and increased the abundance of beneficial bacteria

A total of 404 operational taxonomic units were obtained from the four sample groups, of which 333 were shared among all groups (Figure 4A). The gut microbiota composition of each group was ranked in descending order of abundance at the phylum and genus levels, as shown in Figures 4B,C.

**Figure 4:**
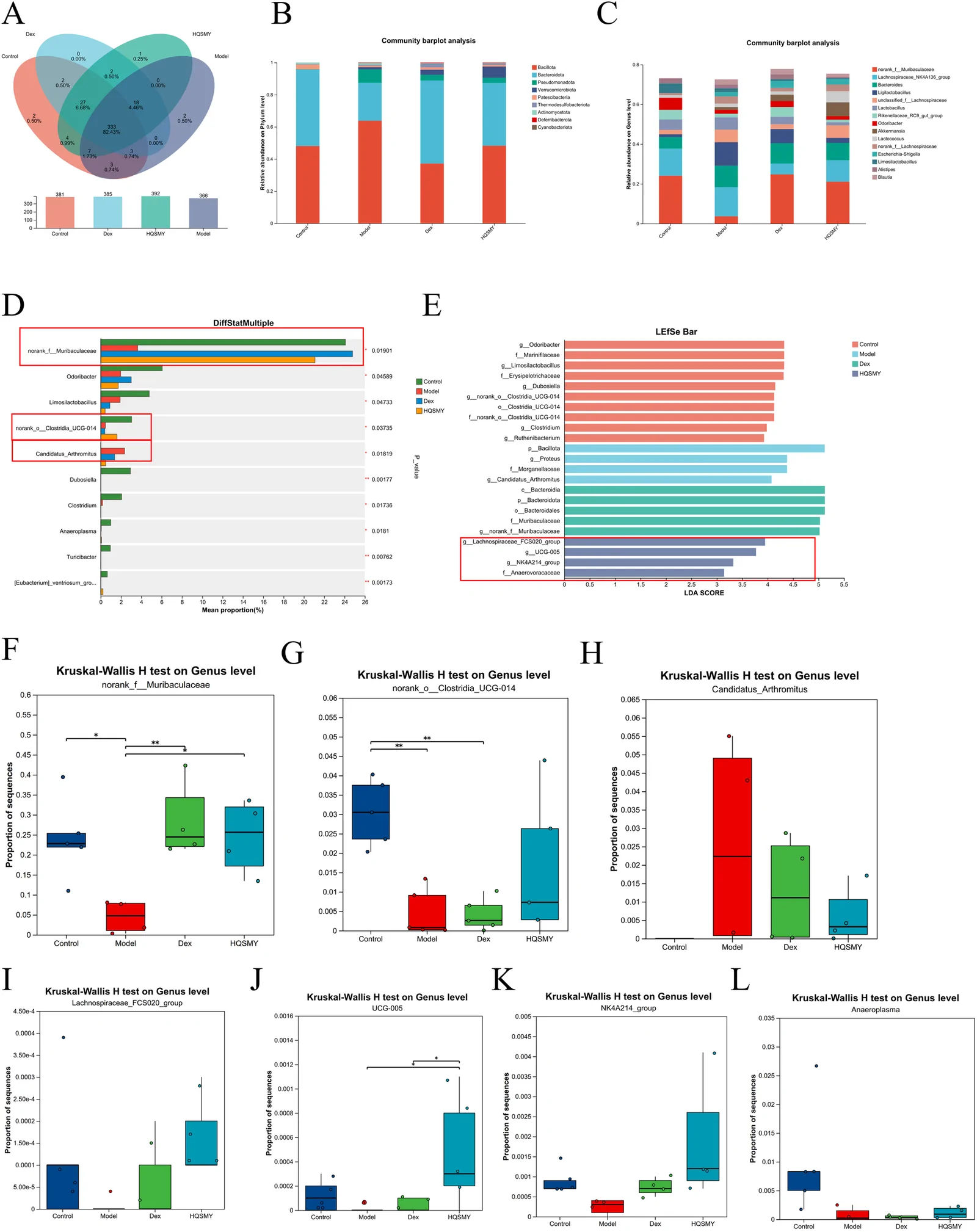
Community structure and species-level differential analysis. **(A)** OTU sample network diagram between groups. **(B)** Phylum-level bacterial composition. **(C)** Genus-level bacterial composition. **(D)** Multiple group difference test. **(E)** Lefse multistage species difference discriminant analysis. **(F)** norank_f_Muribaculaceae. **(G)** norank_o_Clostridia_UCG-014. **(H)** Candidatus Arthromitus. **(I)** Lachnospiraceae_FCS020_group. **(J)** UCG_005. **(K)** NK4A214_group. **(L)** Anaeroplasma.

At the phylum level, compared to the Model group, HQSMY increased the relative abundance of Bacteroidota and Verrucomicrobiota (Figure 4B). At the genus level, HQSMY increased the relative abundance of norank_f_Muribaculaceae (a genus-level unclassified group within the family Muribaculaceae) and decreased Lachnospiraceae_NK4A136_group, Lactobacillus, and Ligilactobacillus (Figure 4C).

Figures 4D,E present the relative abundance and intergroup differences of microbial taxa via stacked bar charts and LEfSe multi-level species differential analysis, respectively. Compared with the Model group, the HQSMY group exhibited increased abundance of norank_f_Muribaculaceae (family Muribaculaceae, reported at the genus level), norank_o_Clostridia_UCG-014 (order level), Lachnospiraceae_FCS020_group (genus), UCG_005 (genus), NK4A214_group (genus), and Anaeroplasma (genus), while showing decreased abundance of Candidatus_Arthromitus (genus). These microbial taxa may represent the core bacterial species involved in the therapeutic effects of HQSMY treatment for AIC. Figures 4F–L present box plots illustrating differences in these taxa across groups.

#### 4.3.3 HQSMY restored the amino acid and energy metabolism activities in model group to control group

The PICRUSt2 functional prediction enables identification of microbial community functions, including metabolic pathways, biochemical pathways, and orthologous groups involved in disease processes. Figure 5A shows the functional classification of protein sequences in the HQSMY sample. The results indicated that expression levels were relatively high in categories such as amino acid transport and metabolism, carbohydrate transport and metabolism, translation, ribosomal structure and biogenesis, and transcription. In multiple pathways, HQSMY mitigated metabolic pathways activated in the Model group, restoring them to Control group levels. Figure 5B shows a heatmap of KEGG pathway level 2, where HQSMY affected pathways including carbohydrate metabolism, amino acid metabolism, and energy metabolism. Combined with deeper (Level 3) KEGG information, purine metabolism, amino sugar and nucleotide sugar metabolism, quorum sensing, and glycolysis/gluconeogenesis pathways may be involved in energy metabolism and intestinal barrier function (Figure 5C). In the MetaCyc pathways, HQSMY also downregulated energy metabolic pathways in gut microbes, such as glycolysis III, as well as the pentose phosphate pathway. Additionally, HQSMY exerted inhibitory effects on nucleotide metabolism, amino acid metabolism, and lipid metabolism of the microbial community, which helped to better maintain gut homeostasis, such as adenosine deoxyribonucleotides *de novo* biosynthesis II, L-isoleucine biosynthesis I, and CDP-diacylglycerol biosynthesis I (Figure 5D).

**Figure 5:**
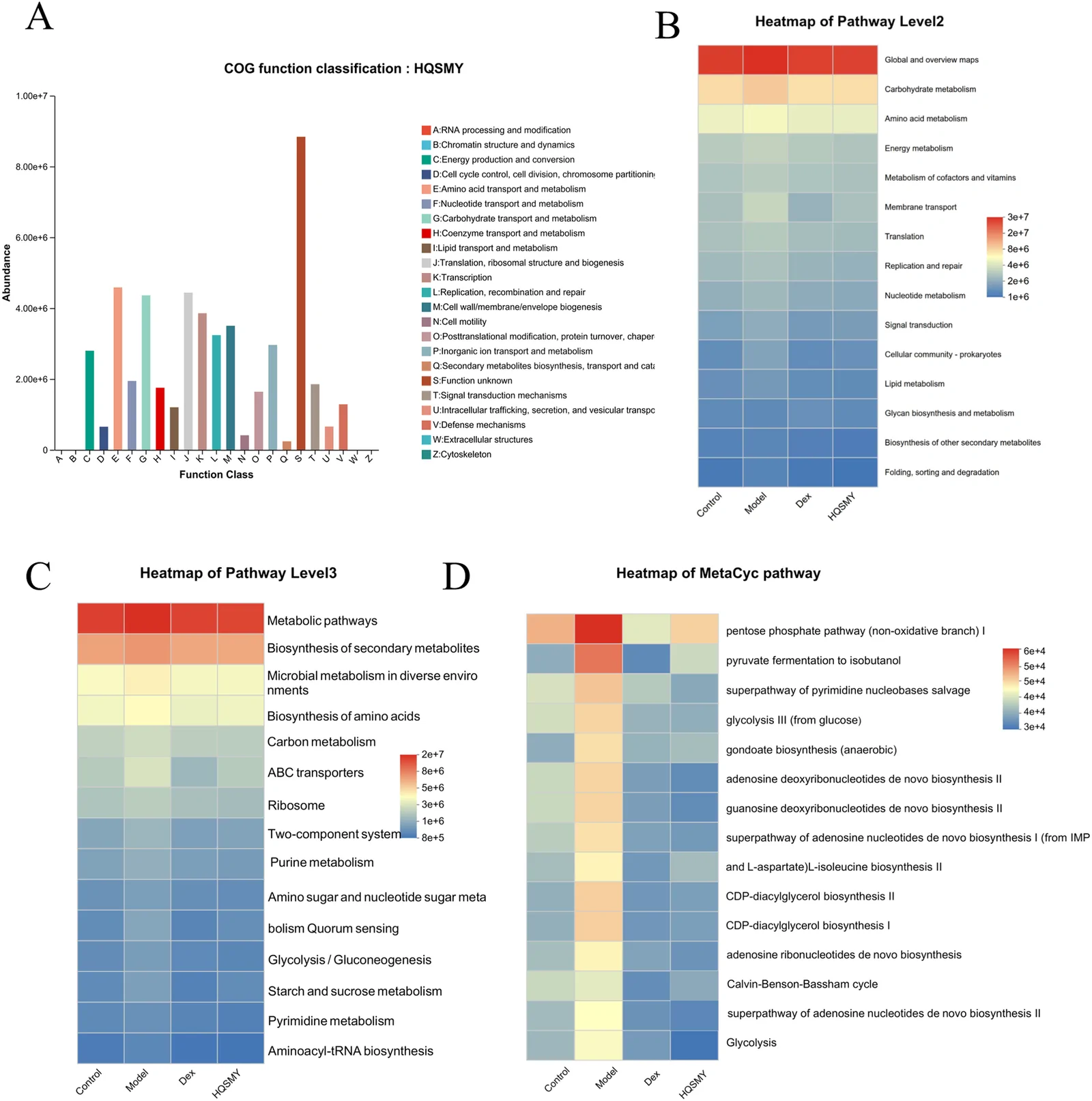
PICRUSt2 function prediction analysis. **(A)** The function classification of protein sequences in the HQSMY sample. **(B)** Heatmap of KEGG pathway level 2. **(C)** Heatmap of KEGG pathway level 3 (20–22). **(D)** Heatmap of MetaCyc pathways.

### 4.4 Prediction of HQSMY's mechanism for treating AIC based on network pharmacology analysis

#### 4.4.1 Target screening and enrichment analysis

Based on the aforementioned 42 bioactive compounds, a “herb-compound-target” network was constructed (Figure 6A). Using breast cancer, AIC, and drug targets as queries, 121 intersecting targets were identified (Figure 6B). Figure 6C shows the protein-protein interaction network, containing 121 nodes and 2,440 edges. Core targets included AKT1, HIF1A, ESR1, IL-6, BCL2, JUN, HSP90AA1, STAT3, and CCND1 (Figure 6D).

**Figure 6:**
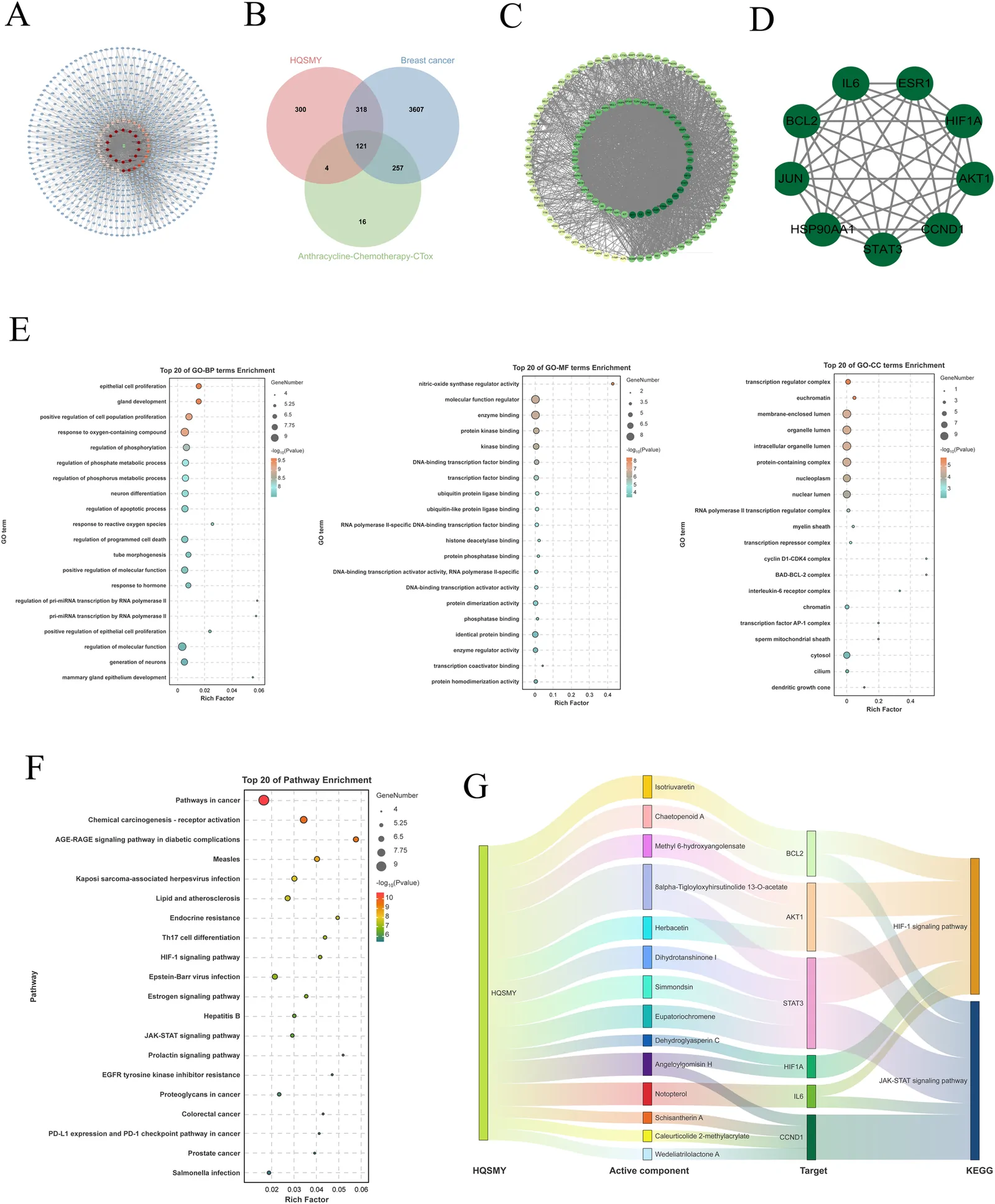
Target screening and enrichment analysis. **(A)** 42 blood active ingredients and the “herb-compound-target” network of targets. **(B)** Venn diagram. **(C)** PPI diagram. **(D)** Network diagram of 9 key targets. **(E)** GO analysis. **(F)** KEGG analysis (20–22). **(G)** A Sankey diagram illustrating the core genes and active components associated with the HIF-1 and JAK-STAT signaling pathways.

Functional enrichment analysis was performed on these 9 key target genes. Figure 6E illustrates the GO enrichment analysis, including Biological Process (BP), Molecular Function (MF), and Cellular Component (CC). GO-BP analysis indicated that HQSMY may affect energy metabolism through multiple biological processes, including positive regulation of cell population proliferation, response to oxygen-containing compounds, regulation of phosphorylation, response to reactive oxygen species, and regulation of pri-miRNA transcription by RNA polymerase II. GO-MF analysis revealed that HQSMY can regulate molecular functions such as nitric-oxide synthase regulator activity, protein phosphatase binding, ubiquitin protein ligase binding, and RNA polymerase II-specific DNA-binding transcription factor binding, which might indirectly influence gut microbiota. Additionally, GO-CC analysis suggested regulation of the cell cycle and inflammatory response through cellular components including the cyclin D1-CDK4 complex, BAD-BCL-2 complex, and interleukin-6 receptor complex. Figure 6F displays the top 20 enriched KEGG pathways, including lipid and atherosclerosis, Th17 cell differentiation, HIF-1 signaling pathway, and JAK-STAT signaling pathway. After an extensive review of the literature and considering the functional enrichment analysis (23–26), we predicted the HIF-1 and JAK-STAT signaling pathways as key pathways for HQSMY treatment of AIC. A Sankey diagram was generated using core genes and active components corresponding to the HIF-1 and JAK-STAT signaling pathways (Figure 6G).

The core targets predicted through the dendrogram were AKT1, HIF1A, IL-6, BCL2, STAT3, and CCND1. The active ingredients were isotriuvaretin, chaetopenoid A, methyl 6-hydroxyangolensate, 8alpha-tigloyloxyhirsutinolide 13-O-acetate, herbacetin, dihydrotanshinone I, simmondsin, eupatoriochromene, dehydroglyasperin C, angeloylgomisin H, notopterol, schisantherin A, caleurticolide 2-methylacrylate, and wedeliatrilolactone A. Subsequently, we performed molecular docking to validate target-component binding affinity and refined selections based on binding energy.

#### 4.4.2 Validation of the JAK-STAT and HIF-1 signaling pathways

Molecular docking was performed between the six selected core targets and their corresponding active components, and all binding energies were ≤ −5.0 kcal/mol. Basic information on the molecular docking between HQSMY's main components and target proteins is presented in Table 2, where the key interactions included hydrogen bonds. The core components were dihydrotanshinone I, isotriuvaretin, dehydroglyasperin C, schisantherin A, methyl 6-hydroxyangolensate, and notopterol. The docking poses are shown in Figure 7A. Western blot analysis was performed to detect key proteins in the JAK-STAT and HIF-1 pathways. HQSMY treatment downregulated the expression of JAK1 and HIF-1*α* compared to the Model group. STAT3 expression was elevated in the Model group compared to Control, but the decrease after HQSMY treatment did not reach statistical significance.

**Table 2:** Basic information on principal component and target protein molecular docking.

Targets	Molecular name	Sites	Binding energy (kcal/mol)
STAT3	Dihydrotanshinone I	ASP-369, HIS-437, LEU-438, THR-440	−8.8
BCL2	Isotriuvaretin	VAL-133	−8.4
HIF1A	Dehydroglyasperin C	ASP-256, GLU-257, HIS−378	−7.4
CCND1	Schisantherin A	ARG-26, LYS-33	−7.3
AKT1	Methyl 6-hydroxyangolensate	LYS-14, GLY-16, LYS-17, ARG−25	−6.8
IL6	Notopterol	ARG-169	−5.4

**Figure 7:**
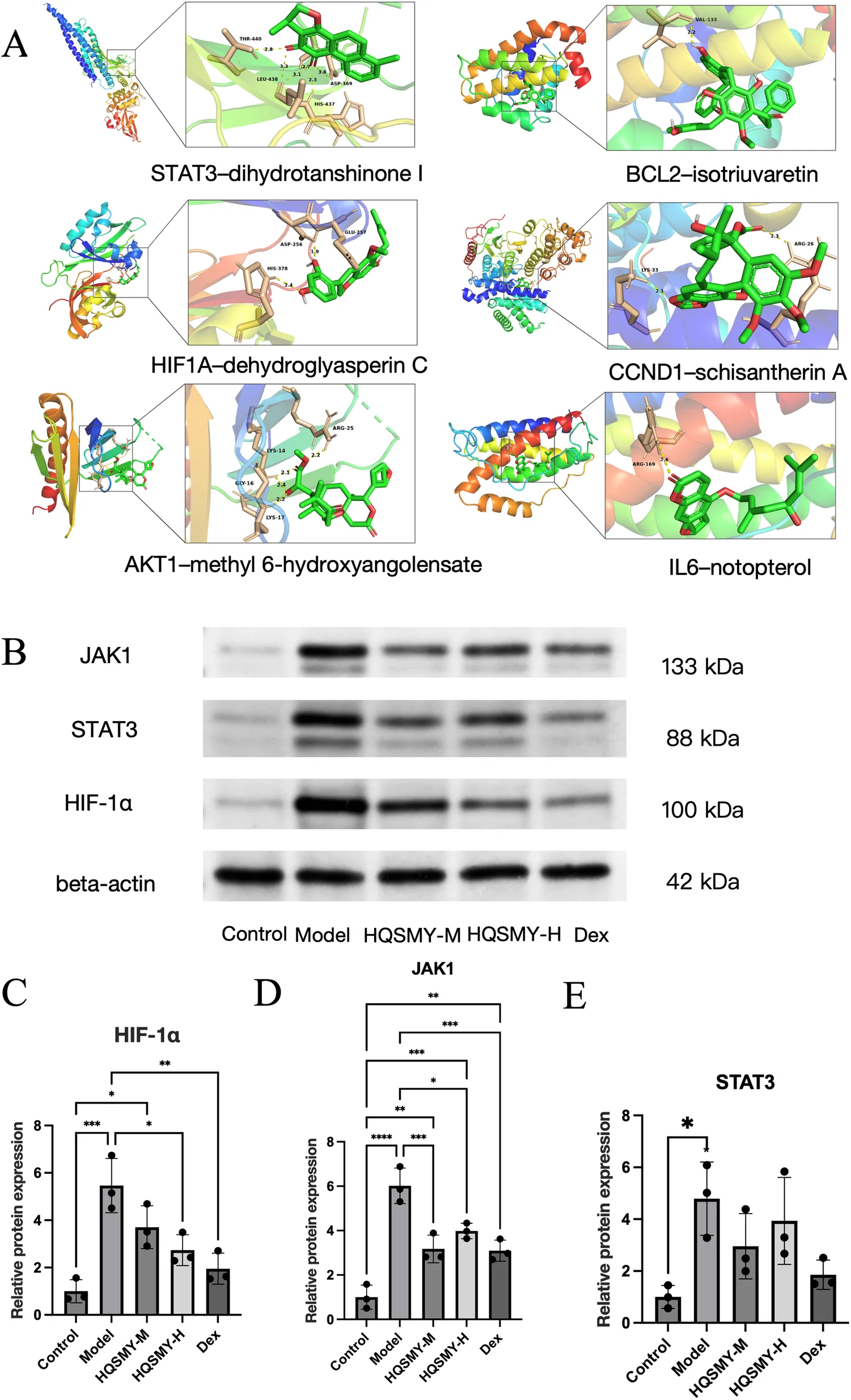
Molecular docking of HQSMY active components with target proteins and experimental validation of the JAK-STAT and HIF-1 pathways in cardiac tissue. **(A)** Molecular docking patterns of six active components with their corresponding targets. The 3D binding modes are shown as global views (left) and local amplified views (right) for each pair (from left to right, top to bottom): STAT3–dihydrotanshinone I, BCL2–isotriuvaretin, HIF1A–dehydroglyasperin C, CCND1–schisantherin A, AKT1–methyl 6-hydroxyangolensate, and IL6–notopterol. **(B)** Representative Western blot images of JAK1, STAT3, HIF-1*α*, and *β*-actin. This experiment was repeated independently three times with similar results. **(C–E)** Quantitative analysis of HIF-1*α*, JAK1, and STAT3 protein expression normalized to β-actin. Data are presented as individual values with mean ± SD (n = 3 independent experiments). **P < 0.01 vs. the Control group; ^##^*P* < 0.01 vs. the Model group (one-way ANOVA followed by Tukey's *post-hoc* test).

## 5 Discussion

AIC is triggered by multiple complex mechanisms, including oxidative stress, inflammation, autophagy, apoptosis, and energy metabolism disorder (27). Among these, inflammation is considered a fundamental cause of AIC progression. Excessive and prolonged inflammation leads to tissue destruction and progressive fibrosis, impairing cardiac function (28, 29). According to TCM theory, “The turbid qi returns to the heart, and the excessive essence pervades the blood vessels.” This concept is consistent with the fact that anthracyclines not only affect the heart but also damage the intestinal barrier, promoting the translocation of endotoxins and microbes into systemic circulation, thereby causing systemic inflammation and cardiovascular complications (30). Notably, a study showed that fecal microbiota transplantation from Dox-treated mice to germ-free mice induced cardiotoxicity, highlighting the critical role of gut microbiota dysbiosis in AIC (31).

Based on the association between gut microbiota and cardiovascular health (32), protecting the gut microbiota may represent a promising strategy to mitigate cardiovascular complications from chemotherapy. The bioactive components of HQSMY are absorbed into the bloodstream via the intestinal barrier following oral administration. This study demonstrated that HQSMY restored *α*-diversity, exhibited a higher microbial health index, and a lower dysbiosis index. Combined with principal coordinate analysis, these findings suggested that the beneficial effect of HQSMY on intestinal flora was equivalent to or superior to that of Dex. Members of the phylum Bacteroidota (formerly Bacteroidetes) are key contributors to intestinal homeostasis (33). At the phylum level, HQSMY increased the abundance of Bacteroidota and restored the abundance of Verrucomicrobiota while decreasing that of Bacillota. At the genus level, HQSMY increased the abundance of norank_f_Muribaculaceae and decreased Lachnospiraceae_NK4A136_group, Lactobacillus, and Ligilactobacillus. Taxonomic differential analysis revealed that HQSMY increased the abundance of norank_f_Muribaculaceae, norank_o_Clostridia_UCG-014, Lachnospiraceae_FCS020_group, UCG_005, NK4A214_group, and Anaeroplasma, while decreasing that of Candidatus_Arthromitus. These microbial taxa may represent the core bacterial groups involved in the therapeutic effects of HQSMY for AIC.

Members of the family Muribaculaceae have a strong capacity to metabolize endogenous (mucin glycans) and exogenous (dietary fibre) polysaccharides, can produce SCFAs, and have cross-feeding relationships with Bifidobacterium and Lactobacillus (34). An increased abundance of Muribaculaceae modulates lipid metabolism and improves cardiomyocyte survival rates (35). Functional prediction analysis indicated that HQSMY restored multiple intestinal microbial metabolic pathways to normal levels, focusing on energy metabolism and the synthesis and transport of amino acids and fatty acids. This may be linked to the production of acetate, propionate, and butyrate (36). Notably, SCFAs can suppress lipopolysaccharide-induced inflammation by inhibiting inducible nitric oxide synthase activity (37).

The JAK-STAT pathway is recognized as a central signaling hub in cellular communication. In cardiovascular diseases, inhibiting this pathway can alleviate inflammation and myocardial fibrosis (24, 38). Butyrate enhances H3K27ac enrichment around the SOCS1 gene promoter region, thereby increasing SOCS1 protein expression and ultimately suppressing JAK-STAT signaling activation (39). HQSMY normalized energy metabolic pathways in gut microbes, such as glycolysis III and the pentose phosphate pathway. Increased microbial oxygen consumption and inflammation caused by dysbiosis jointly lead to local intestinal hypoxia and disrupt the integrity of the intestinal barrier (40). Furthermore, IL-6 activates the JAK-STAT signaling pathway, particularly STAT3, playing a crucial role in Th17 cell differentiation and inflammatory responses in the myocardium (41). Systemic inflammation driven by gut dysbiosis may exacerbate this local inflammatory cascade in cardiac tissue (42, 43).We hypothesize that anthracycline-induced gut dysbiosis may increase intestinal permeability and facilitate LPS translocation, thereby contributing to myocardial inflammatory responses and HIF-1*α* activation. HQSMY restored multiple microbial metabolic pathways, including glycolysis and amino acid metabolism, which may be associated with improved intestinal barrier integrity and reduced systemic inflammatory burden. Consequently, the decreased inflammatory milieu in the myocardium may contribute to the attenuation of HIF-1*α* stabilization observed in cardiac tissue (Figure 7). However, direct evidence connecting specific microbial taxa or metabolites to cardiac HIF-1*α* regulation was not established in the present study.

In conclusion, the components of HQSMY may exert anti-inflammatory effects in myocardial tissue, while concomitantly altering gut microbiota composition. These changes may be associated with increased SCFA production, thereby contributing to intestinal barrier protection and attenuation of LPS-induced inflammation. It should be noted that compound identification was based on database matching of MS/MS spectra and retention times; confirmation with reference standards was not performed. Although these findings suggest a potential association between gut microbiota modulation and cardiac protection, a direct causal relationship was not established. The relatively small sample size may compromise the robustness of the findings. Future studies will prioritize GC-MS-based SCFA quantification and multi-omics integration to further elucidate the mechanisms of HQSMY in AIC. It should be noted that histological assessment was performed on residual cardiac tissue sections; unbiased whole-section evaluation was not feasible due to sample availability. Direct measurement of SCFA concentrations was not performed; the association between HQSMY and SCFA production was inferred from changes in the abundance of SCFA-producing bacterial taxa.

## Data Availability

The datasets presented in this study can be found in online repositories. The names of the repository/repositories and accession number(s) can be found in the article/Supplementary Material.
